# Identification of the molecular determinants of antagonist potency in the allosteric binding pocket of human P2X4

**DOI:** 10.3389/fphar.2023.1101023

**Published:** 2023-02-09

**Authors:** Gaia Pasqualetto, Marika Zuanon, Andrea Brancale, Mark T. Young

**Affiliations:** ^1^ School of Biosciences, Cardiff University, Cardiff, United Kingdom; ^2^ School of Pharmacy and Pharmaceutical Sciences, Cardiff University, Cardiff, United Kingdom; ^3^ Department of Organic Chemistry, Vysoká škola chemicko-technologická v Praze, Prague, Czechia

**Keywords:** P2X4, BX430, allosteric antagonist, structure-function, calcium channel

## Abstract

P2X receptors are a family of ATP-gated cation channels comprising seven subtypes in mammals, which play key roles in nerve transmission, pain sensation and inflammation. The P2X4 receptor in particular has attracted significant interest from pharmaceutical companies due to its physiological roles in neuropathic pain and modulation of vascular tone. A number of potent small-molecule P2X4 receptor antagonists have been developed, including the allosteric P2X4 receptor antagonist BX430, which is approximately 30-fold more potent at human P2X4 compared with the rat isoform. A single amino-acid difference between human and rat P2X4 (I312T), located in an allosteric pocket, has previously been identified as critical for BX430 sensitivity, implying that BX430 binds in this pocket. Using a combination of mutagenesis, functional assay in mammalian cells and *in silico* docking we confirmed these findings. Induced-fit docking, permitting the sidechains of the amino-acids of P2X4 to move, showed that BX430 could access a deeper portion of the allosteric pocket, and that the sidechain of Lys-298 was important for shaping the cavity. We then performed blind docking of 12 additional P2X4 antagonists into the receptor extracellular domain, finding that many of these compounds favored the same pocket as BX430 from their calculated binding energies. Induced-fit docking of these compounds in the allosteric pocket enabled us to show that antagonists with high potency (IC_50_ ≤ 100 nM) bind deep in the allosteric pocket, disrupting a network of interacting amino acids including Asp-85, Ala-87, Asp-88, and Ala-297, which are vital for transmitting the conformational change following ATP binding to channel gating. Our work confirms the importance of Ile-312 for BX430 sensitivity, demonstrates that the allosteric pocket where BX430 binds is a plausible binding pocket for a series of P2X4 antagonists, and suggests a mode of action for these allosteric antagonists involving disruption of a key structural motif required for the conformational change induced in P2X4 when ATP binds.

## 1 Introduction

P2X receptors are eukaryotic ligand-gated cation channels activated by extracellular ATP. There are seven receptor subtypes in mammals, which combine into homo- or hetero-trimers, with differing tissue expression and pharmacological properties (North, 2016). P2X receptors play numerous important physiological roles in mammals, including nerve transmission, pain sensation, inflammation, and higher neural functions, and as such are important drug targets (North and Jarvis, 2013). There is a significant body of evidence from animal models that implicates the P2X4 receptor in neuropathic pain (Tsuda et al., 2003; Matsumura et al., 2016). Furthermore, evidence from both animal models and human mutation studies link P2X4 to the regulation of vascular tone and blood pressure (Yamamoto et al., 2006; Stokes et al., 2011). Due to their roles in human disease, there has been significant interest in developing potent P2X4 receptor modulators and the determination of the crystal structures of a series of P2X receptors including zebrafish P2X4 (Hattori and Gouaux, 2012) and giant panda P2X7 (Karasawa and Kawate, 2016) has proven pivotal in the understanding of ligand binding and receptor activation. In particular, the recent and surprising discovery that a series of P2X7 antagonists (previously thought to be competitive) bound to a site distinct from the ATP binding site delineated an important allosteric binding pocket in P2X receptors.

**FIGURE 1:**
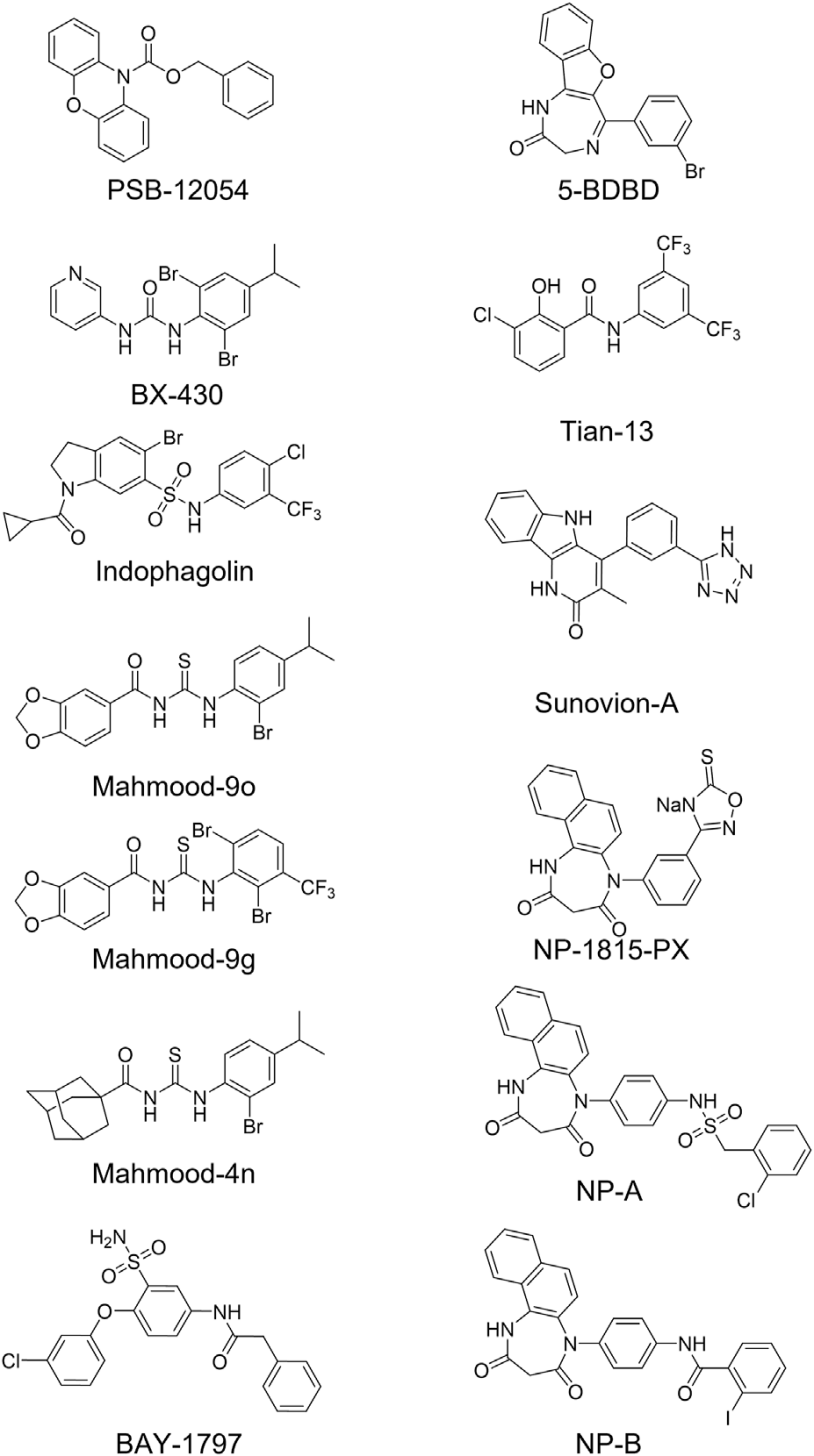
Structures of potent and selective P2X4 antagonists reported to date. In this study, two of the most potent NipponChemipharm (NP) compounds series have been arbitrarily named NP-A and NP-B, the compound patented by Souvion is referred to as Souvion-A. Compound 4n [Mahmood et al. (2022a)] and compounds 9g and 9o [Mahmood et al. (2022b)] are referred to as Mahmood 4n, Mahmood 9g, and Mahmood 9o. Compound 13 (Tian M. et al., 2020) is referred to as Tian 13.

Among the P2X4 antagonists, some are reportedly allosteric, including 5-BDBD, which displays an IC_50_ of 0.5 μM at human P2X4 (Fischer et al., 2004). Other reported antagonists with potencies in the low micromolar range are PSB-12054 and PSB-12062 (Hernandez-Olmos et al., 2012) although these compounds only display partial solubility. In 2013, Sunovion Pharmaceutical Inc. Patented a series of compounds (including the compound herein arbitrarily named Sunovion-A, Figure 1) displaying high potencies (IC_50_ < 100 nM) (Newcom and Spear, 2013). Two years later, Ase et al. (2015) reported the characterization of BX430, which displayed interesting and unexpected P2X4 species-selective potencies. Despite a lower amino acid sequence similarity between human P2X4 and zebrafish P2X4 receptor, BX430 potency was similar at human P2X4 and zebrafish P2X4 (IC_50_ values of 0.5 and 1.89 μM, respectively) compared to its very weak activity against the rat and mouse orthologous (IC_50_ values both >10 μM).

Other potent antagonists have been reported since, including a series developed by Nippon Chemipharm (NP-1815-PX, NP-A and NP-B) with submicromolar potency (IC_50_ values approx. 0.25 μM at human, rat and mouse) that displayed an anti-allodynic effect in an *in vivo* rodent model of injury-induced allodynia (Matsumura et al., 2016; Ushioda et al., 2020). In 2019, BAY-1797 was identified as a P2X4 antagonist with a similar potency across human, rat and mouse P2X4 orthologues (IC_50_ of 100 nM at rat and human and 200 nM at mouse) (Werner et al., 2019). Whilst designing P2X1 antagonists, (Tian M. et al., 2020) synthesized a series of compounds (including the compound with structure reported in Figure 1, here referred to as “Tian-13”) with nanomolar potency at P2X4 but with only partial selectivity across the receptor family (P2X1 IC_50_ 58 ± 21.9 nM; P2X4 IC_50_ 48.8 ± 11 nM and P2X7 IC_50_ 177 ± 11 nM). Furthermore, an interesting approach was employed by Carnero Corrales et al. (2021), who identified, *via* thermal proteome profiling, P2X4 as potential target of indophagolin. Finally, Mahmood et al. (2022a); Mahmood et al. (2022b) recently reported several series of compounds (including series 4 and 9) with submicromolar potency at human P2X4. Figure 1; Table 1 summarize the chemical structures and reported IC_50_ values of the P2X4 antagonists considered in this study.

**TABLE 1:** Reported potencies for known P2X4 antagonists. n.r, Not reported. ^a^ Fischer et al. (2004); ^b^ Abdelrahman et al. (2017); ^c^ Sophocleous et al. (2020); ^d^ Coddou et al. (2019); ^e^ Werner et al. (2019); ^f^ Ase et al. (2015); ^g^ Carnero Corrales et al. (2021); ^h^ Mahmood et al. (2022a); ^i^ Mahmood et al. (2022b); ^j^ Hernandez-Olmost et al. (2012); ^k^ Matsumura et al. (2016), and ^l^ Ushioda et al. (2020) ^m^ Newcom and Spear (2013); ^n^ Tian et al. (2020).

Compound	hP2X4 IC_50_	rP2X4 IC_50_	mP2X4 IC_50_
5-BDBD	0.5 μM^a^; 0.348 μM^b^; 5.24 μM^c^	3.47 μM^b^; 0.75 μM^d^	2.04 μM^b^
BAY-1797	0.1 µM^e^	0.1 µM^e^	0.2 µM^e^
BX-430	0.54 μM^f^; 1.94 μM^c^	>10 µM^f^	>10 µM^f^
Indophagolin	0.140 μM^g^	n.a	n.a
Mahmood-4n	0.04 μM^h^	n.r	n.r
Mahmood-9g	0.055 μM^i^	n.r	n.r
Mahmood-9o	0.039 μM^i^	n.r	n.r
NP-1815-PX	0.26 µM^k, l^	n.r	n.r
NP-A	0.064 μM^k, l^	n.r	n.r
NP-B	0.43 μM^k, l^	n.r	n.r
PSB-12054	0.19 μM^j^	2.1 µM^j^	1.8 µM^j^
Sunovion-A	<0.1 μM^m^	n.r	n.r
Tian-13	0.049 µM^n^	n.r	n.r

We initially hypothesized that BX430 might bind to the equivalent site on P2X4 to that recently identified in P2X7. We constructed a molecular model of human P2X4 based on the zebrafish P2X4 crystal structure (Hattori and Gouaux, 2012), used molecular docking to ascertain that BX430 was able to bind to the allosteric pocket, and identified a single amino-acid difference between human and rat P2X4 in the allosteric pocket (I312T) previously reported to be responsible for differential BX430 potency (Ase et al., 2019). Functional assays using calcium influx in stably transfected 1321N1 astrocytoma cells confirmed the difference between BX430 potency at human P2X4 wild type and rat P2X4 wild type, whilst the single amino-acid mutation of either human P2X4 (I312T) or rat P2X4 (T312I) was sufficient to confer the BX430 sensitivity of the other receptor orthologue, demonstrating that BX430 binds at this allosteric site in the receptor. Our results are in very close agreement with those of Ase et al. (2019) who used patch clamp electrophysiology and molecular docking studies, and reinforce their findings using an independent assay. The work of Ase et al. (2019) and our data demonstrate that BX430 binds to the equivalent allosteric site in P2X4 to that accessed by several allosteric P2X7 antagonists.

We next hypothesized that other P2X4 antagonists may bind in the same allosteric pocket as BX430 and employed *in silico* induced-fit docking approaches (where the amino-acid sidechains of the pocket are allowed to move) with a total of 12 compounds, identifying the ligand-protein interactions within the allosteric pocket important for high-potency antagonism. Using this approach, we were able to show that a network of interactions with Arg-82, Asp-85, Ala-87, Asp-88, Trp-164, Ala-297, Glu-307, and Arg-309, deep within the allosteric pocket and critical for efficient P2X4 gating, are likely to be disrupted by antagonist binding. This data will be useful for the design and development of potent P2X4 antagonists in future.

## 2 Materials and methods

### 2.1 Plasmid generation

Plasmid encoding rat P2X4 (r4-WT) (Royle et al., 2005) was a kind gift from Ruth Murrell-Lagnado, whilst that encoding human P2X4 with a C-terminal (His)_10_ tag has been reported previously (Young et al., 2008). Plasmids containing rat P2X4 T312I (r4-T312I) and human P2X4 I312T mutants (h4-I312T) were generated using the QuikChange Lightning site-directed mutagenesis kit (Agilent) according to manufacturer’s instructions. Sequencing of the constructs and of the genomic DNA from stably transfected cells confirmed correct full coding sequences.

### 2.2 Development and selection of 1321N1 cell lines stably expressing human and rat P2X4 receptors

1321N1 astrocytoma cells were cultured (at 37°C, 5% CO2) in Dulbecco’s modified Eagle’s medium (DMEM/F-12 with Glutamax) supplemented with 10% foetal bovine serum (FBS) and 200 unit/mL of penicillin and streptomycin antibiotics (Fisher Scientific). Stably transfected clones were grown in DMEM/F-12 with Glutamax medium supplemented with G-418 (Geneticin^®^, Fisher Scientific), 600 μg/mL and 150 μg/mL during selection and maintenance, respectively. FuGene HD Transfection Reagent (Promega) was used according to the manufacturer’s protocol for the generation of stable cell line expressing h4-WT, r4-WT, h4-I312T, and r4-T312I. Each cell line was then tested for P2X4 receptor expression by Western blot and for functionality using the Ca^2+^ influx assay.

### 2.3 Calcium influx measurements

Cells were plated the day before assay into poly-lysine-coated 96-well plates at 40–55,000 cells/mL. To load cells with calcium-sensitive fluorescent dye, culture medium was replaced with modified Ringer’s buffer (140 mM NaCl, 10 mM HEPES, 10 mM Glucose, 1 mM MgCl_2_, 1 mM CaCl_2_, 2.5 mM KCl, 0.5% BSA, pH = 7.4) containing 2.6 μM FLUO4-AM (Fisher Scientific) and 250 μM probenecid (Sigma), and cells were incubated for 20–30 min. Prior to assay, buffer was replaced with fresh modified Ringer’s buffer containing probenecid but not the dye. BX430 was incubated at increasing concentrations (0.03 μM–100 μM) for approximately 30 min prior to eliciting ATP responses. The final DMSO concentration did not exceed 0.1%. A Fluoroskan Ascent FL plate reader (Fisher Scientific) equipped with a solution dispenser and an appropriate filter pair (excitation: 485 nm, emission: 538 nm) was used to record 5-s baseline followed by P2X4-mediated calcium influx measurements for 20–25 s after ATP stimulation (final concentration ranging between 0.03 and 100 μM in buffered solution, pH = 7.4). The collected responses were normalized to the first data-point recorded after ATP injection. The amplitude of ATP responses was calculated as ΔF/F_0_, where ΔF = F_1_−F_0_, subtracting the fluorescent background as suggested by Bootman et al. (2013).

### 2.4 Homology modelling and ligand docking

*In silico* simulations were performed on a MAC pro 2.80 GHz Quad-core Intel Xeon running Ubuntu 12.04 LTS. Graphical representations of molecular structures were generated using MOE (Molecular Operating Environment (MOE), 2019.01; Chemical Computing Group ULC, Montreal, Canada). All crystal structure files used were retrieved from the RCSB Protein Data Bank (http://www.rcsb.org) as PDB file format and protonated with MOE. The human P2X4 receptor homology model was generated using MOE 2019.01 using AMBER10:EHT force field and standard parameters for the single template mode. The model was checked visually and via PROCHECK (Laskowski et al., 1993) to exclude gross errors in protein geometry. Blind docking simulations were performed on the extracellular domain using Achilles Blind Docking Server (https://bio-hpc.ucam.edu/achilles/) (Sanchez-Linares et al., 2012) and ligand MOL2 files pre-generated using MOE. Docking with Glide involved pre-processing of the receptor structure *via* the built-in Schrodinger tools Prep Wiz, Epik, Impact and Prime (Schrödinger, New York). Ligands were prepared with LigPrep using the OPLS_2005 force field. In simulations where the protein was treated as rigid body, a docking grid was generated with set coordinates corresponding to the mid-point of the cavity and box size of 18 Å^3^ and docking simulations were run with Glide Extra Precision (XP) protocol using standard parameters. In induced-fit docking simulations, Glide Standard Precision (SP) was applied generating 20 poses in the first stage before sidechain optimization within 5 Å from the ligand using Prime, followed by Glide re-docking into structures within 30.0 kcal/mol of the best structures (top 20) with SP precision.

### 2.5 Data analysis and statistics

Data processing and data analysis were carried out using Graphpad Prism version 6 for Mac. Figures are presented as Mean ± SEM. Statistical significance of any difference observed between samples was calculated through one-way ANOVA followed by Dunnett’s multiple comparisons test with a single pooled variance (control, unless otherwise specified) or, when comparing only two sets of data, using an unpaired *t*-test with Welch’s correction. When comparing data obtained from multiple independent experiments (N), each dataset was normalized to the mean value obtained for the vehicle control. A non-linear regression (curve fit) with four parameters (constraints to Bottom = 0 and Top = 100 were applied when considering normalized ATP concentration-response curves) was used to determine dose-response correlation and calculate EC_50_ and IC_50_ values.

## 3 Results

### 3.1 Docking of BX430 and analysis of human and rat P2X4 sequences in the putative BX430 binding site reveals I312 as a key interacting residue

**FIGURE 2:**
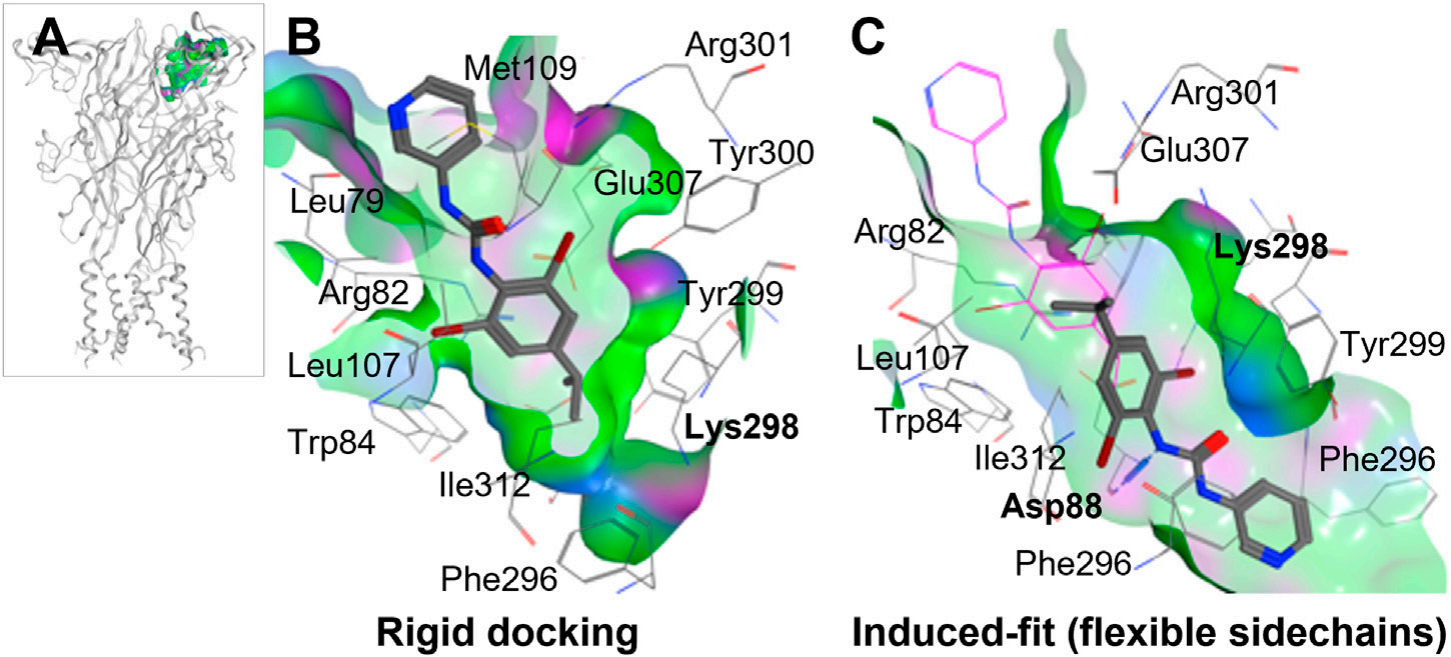
Molecular docking of BX430 into the allosteric pocket of human P2X4. **(A)**. Molecular model of human P2X4 showing the position of the pocket in the extracellular domain. **(B–C)**. Alternate binding poses of BX430 modelled into the allosteric pocket of human P2X4 using rigid **(B)** or induced-fit **(C)** docking.

To perform ligand docking studies, we first generated a molecular model of human P2X4 based upon the crystal structure of *Danio rerio* (zebrafish) P2X4 in the closed state [PDB ID; 4DW0 (Hattori and Gouaux, 2012)]. By comparison of our model with the panda P2X7 crystal structures in complex with allosteric antagonists (Karasawa and Kawate, 2016), we identified the corresponding putative allosteric pocket in human P2X4 (Figure 2A). When compared to panda P2X7 and the rat P2X7 cryo-EM structure in the closed state [no ligand bound, PDB ID; 6U9V (McCarthy et al., 2019)], the pocket in zebrafish P2X4 (4DW0) and in our model (Figure 2B) presents a narrower tunnel. In addition, in both the zebrafish structure and in our model, residues such as Lys-298 formed a “bottleneck” in the cavity.

Our initial molecular docking simulations with BX430 (using a rigid protein structure model) showed that the dibromo-(methylethyl)phenyl moiety was stabilized by hydrophobic interactions including with residue Ile-312, whilst the urea moiety lay in a polar region potentially making H-bond interactions with adjacent residues (Figure 2B). Based on the position of Lys-298 in the crystal structures of panda P2X7 with the antagonists bound, it was reasonable to consider that Lys-298 could be highly flexible, especially when in presence of a ligand binding in proximity. Therefore, we performed induced-fit docking, with initial docking of the ligand with pocket residues as reference (20 ligand poses), followed by minimization of the residues 5 Å around the ligand, re-docking of the ligand in the induced-fit pocket and scoring of the poses. The induced fit protocol resulted in a substantially different conformation of Lys-298 (Figure 2C) with a wider opening of the bottleneck. The obtained BX430 conformation was similar to that reported by Ase et al. (2019) with BX430 buried deeper and the urea moiety establishing interactions with Asp-88 (backbone, Figure 2B). This may indicate an important role of the Lys-298 sidechain in the shaping of the allosteric pocket.

We then took advantage of the reported differential potency of BX430 at human and rat P2X4 [IC_50_ values of 0.54 μM and >10 μM, respectively (Ase et al., 2015)] and aligned the amino-acid sequences of human and rat P2X4 in the region of the putative BX430 binding site to look for any major residue difference. Strikingly, there was only one significant difference at position 312, which is an isoleucine in human P2X4 and a threonine in rat P2X4. Since our docking studies indicated that this amino-acid residue might be involved in BX430 binding, we made the reciprocal mutations, human P2X4 I312T and rat P2X4 T312I, to assess their effect on BX430 potency.

### 3.2 Exchange of residues at position Ile-312 is sufficient to mediate the differential potency of BX430 at human and rat P2X4 in a calcium-influx assay

**FIGURE 3:**
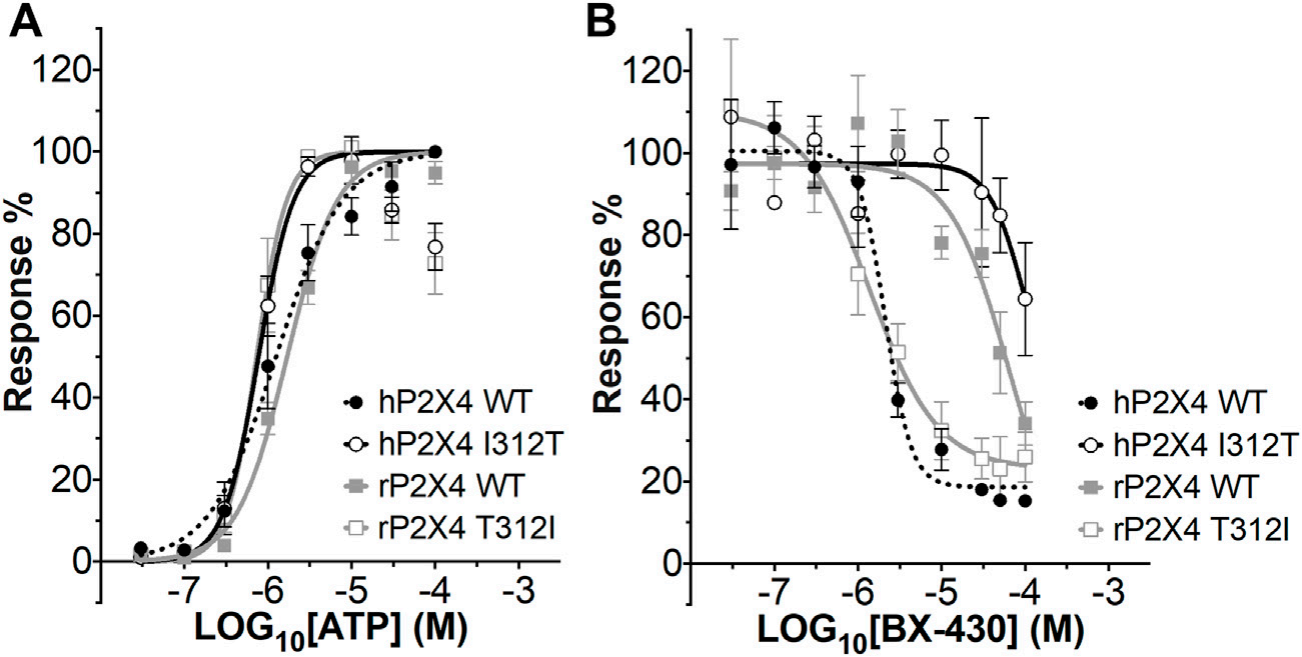
ATP concentration-response and BX430 concentration-inhibition curves for human and rat P2X4 (wild type and mutant) receptors. **(A)**. Mean ATP concentration-response curves of rat P2X4 wild type (rP2X4 WT, grey full squares), rat P2X4 T312I mutant (rP2X4 T312I, gray empty squares), human P2X4 wild type (hP2X4 WT, black full circles), human P2X4 I312T (hP2X4 WT, black empty circles). Responses represent fluorescence increase following ATP addition and were normalized to the maximum response mean value recorded among the concentrations tested. Data merged from 3 or more independent experiments (4–6 technical repeats per experiment). Error bars are SEM. EC_50_ and Hill coefficient values are reported in Table 2. **(B)**. Mean BX430 inhibition curves of rat P2X4 wild type (rP2X4 WT, grey full squares), rat P2X4 T312I mutant (rP2X4 T312I, gray empty squares), human P2X4 wild type (hP2X4 WT, black full circles), human P2X4 I312T (hP2X4 WT, black empty circles). Responses represent fluorescence increase elicited by 1 µM ATP addition after incubation with increasing concentrations of BX430. Data points were normalized to the response obtained with vehicle-only (0.1% DMSO) and are presented as mean ± SEM. Data merged from 2 (for hP2X4 WT) or more independent experiments (3–5 technical repeats per experiment). IC_50_ and Hill coefficient values are reported in Table 3.

We first measured ATP-induced calcium uptake using Fluo-4 in 1321N1 astrocytoma cells stably expressing either wild type human P2X4 (h4-WT), wild type rat P2X4 (r4-WT), human P2X4 I312T (h4-I312T) or rat P2X4 T312I (r4-T312I) receptors (Figure 3A; Table 2). We observed ATP EC_50_ values for h4WT and r4WT of 1.26 ± 0.16 and 1.70 ± 0.30 μM, respectively, consistent with previous studies (Soto et al., 1996; Garcia-Guzman et al., 1997). We also found that the mutations h4-I312T and r4-T312I did not significantly affect ATP peak calcium responses and their EC_50_ was 0.77 ± 0.52 and 0.73 ± 0.61 μM (Table 2), implying that the mutations did not affect the ability of the P2X4 receptors to respond to ATP.

**TABLE 2:** ATP EC_50_, pEC_50,_ and Hill Coefficient values for wild type (WT) and mutant rat and human P2X4 receptors. SEM, standard error of the mean. *indicates a statistically significant difference between rat P2X4 WT and human P2X4 I312T (*p* ≤ 0.05).

Cell line	ATP EC_50_ (µM)	ATP pEC_50_ (± SEM)	Hill coefficient
Human P2X4 WT	1.26	5.901 (± 0.0577)	1.098
Rat P2X4 WT	1.70	5.770 (± 0.0507)*	1.441
Human P2X4 I312T	0.77	6.112 (± 0.028)*	2.057
Rat P2X4 T312I	0.73	6.136 (± 0.0645)	2.475

We next assessed the potency of BX430 in our wild type and mutant cell lines by generating concentration-inhibition curves using EC_50_ concentrations of ATP and estimating the BX-430 IC_50_ at each receptor (Figure 3B; Table 3). We observed IC_50_ values for h4-WT and r4-WT of 2.12 ± 0.60 and 66.1 ± 20 μM (ambiguous fit due to low potency), respectively, consistent with previously published values (Ase et al., 2015). Strikingly, the single point mutation of I312T in human P2X4 was responsible alone for >48-fold decrease in BX-430 potency to a similar value to that of rat P2X4 (IC_50_ 102.4 µM). Conversely, the mutation of T312I in rat P2X4 conferred a BX430 potency similar to that of human P2X4 (IC_50_ 1.4 µM, approx. 47-fold increase), suggesting that the residue at position 312 is sufficient to mediate the differential potency of BX430 at human and rat P2X4 and confirming the results obtained by Ase et al. (2019) obtained *via* patch clamp electrophysiology.

**TABLE 3:** BX430 IC_50_ values for wild type and mutant rat and human P2X4 receptors. SEM, standard error of the mean. *indicates values significantly different to human P2X4 wild type (WT) (*p* ≤ 0.05).

Cell line	BX-430 IC_50_ (µM)	BX-430 pIC_50_ (± SEM)	Hill coefficient
Human P2X4 WT	2.12	5.673 (± 0.0696)	1.098
Rat P2X4 WT	66.1	4.180 (± 0.6884)*	1.441
Human P2X4 I312T	102.4	3.990 (± 2.641)*	2.057
Rat P2X4 T312I	1.4	5.858 (± 0.1575)	2.475

### 3.3 Docking of known allosteric antagonists in the allosteric pocket

We initially explored the hypothesis that some of the recently reported antagonists may bind to the same allosteric pocket, by performing a blind docking simulation. A total of 12 antagonists with low micromolar to nanomolar potencies were docked, including antagonists with biological data demonstrating their binding to the allosteric pocket [BX-430 (Ase et al., 2019) and 5-BDBD (Bidula et al., 2022), which served as benchmark], ligands with previously reported inferred docking [Tian-13 (Tian, M., et al. (2020) and Mahmood 4n and Mahmood 9o (Mahmood et al., 2022a; Mahmood et al., 2022b)] and ligands with no binding information [BAY-1797 (Werner et al., 2019), Mahmood 9g (Mahmood et al., 2022b), NP-1815-PX, NP-A, NP-B (Matsumura et al., 2016; Ushioda et al., 2020)Sunovion-A (Newcom and Spear, 2013), PSB-12054 (Hernandez-Olmos et al., 2012) and indophagolin (Carnero Corrales et al., 2021)]. The Achilles Server simulation estimated binding energies of −6.00 and −7.40 kcal/mol for BX430 and 5-BDBD, respectively for the allosteric pocket. Interestingly, the cavity corresponding to the allosteric pocket was ranked in the Top-5 of favorable cavities for the majority of the ligands (Table 4). Importantly, the allosteric pocket was ranked the Top-1 and Top-5 most favorable cavity for BAY-1797 and NP-A, respectively, with estimated binding energies of −9.50 and −9.30 kcal/mol, suggesting a high probability of binding at this site. Overall, the estimated binding energies showed a good correlation with the respective calculated antagonist IC_50_ with lower values for the more potent compounds (Table 4). Indophagolin and PSB-12054 were the ligands whose blind docking cluster poses scored lowest in the allosteric pocket. These findings suggested that most ligands considered in this study are likely to bind to the same allosteric pocket as BX-430.

**TABLE 4:** Computed binding energies of P2X4 antagonists docked into the human P2X4 allosteric pocket in a blind docking simulation run using the AchillesDock Server. Ranking of the cluster of poses docked in the allosteric pocket (pocket where Ile-312 lies, investigated in this work).

Compound	Binding energy of the cluster (kcal/mol) in hP2X4 allosteric pocket	Rank	Rank after removing redundant clusters
5-BDBD	−7.40	16	8
BAY-1797	−9.50	1	1
BX-430	−6.00	11	5
Mahmood-4n	−7.40	3	3
Mahmood-9g	−7.50	9	8
Mahmood-9o	−7.30	8	5
NP-1815-PX	−9.30	6	6
NP-A	−9.30	6	5
NP-B	−8.70	8	7
PSB-12054	−7.60	13	12
Sunovion-A	−8.90	4	4
Tian-13	n.d	n.d	n.d

**FIGURE 4:**
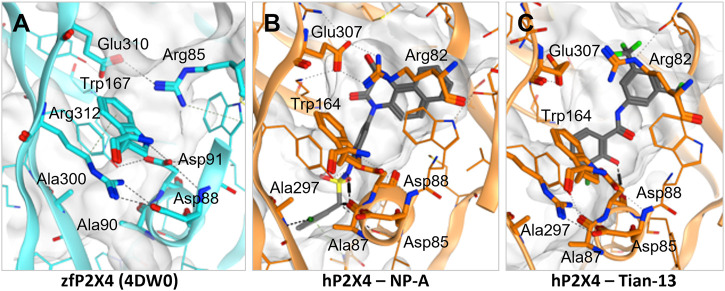
Disruption of the inter-residue protein network by P2X4 antagonists. **(A)**. Crystal structure of zebrafish P2X4 (4DW0) with key residues involved in receptor structural stability and activation (in thick lines). **(B–C)**. Induced-fit docking of NP-A **(B)** and Tian-13 **(C)** into a model of human P2X4. Dashed lines indicate polar interactions.

Induced-fit docking was then used to dock each ligand into the allosteric pocket and investigate the nature of the ligand-target interactions (Figure 4; Supplementary Figure S1). Interestingly, no docking poses within the allosteric pocket were obtained for PSB-12054 *via* the induced fit protocol, suggesting that is unlikely that PSB-12054 and its analogues bind in the same pocket as BX430, at least in the closed state of the receptor. Several key interactions are observed in the allosteric pocket of the zebrafish P2X4 closed-state crystal structure (Figure 4A), including an ionic interaction between Glu-310 and Arg-85 (zebrafish P2X4 numbering). The docking pose of 5-BDBD (Supplementary Figure S1B), was remarkably similar to that previously reported (Bidula et al., 2022), with the amide of the dihydro-2H-benzofuro [3,2-e]-1,4-diazepin-2-one interacting with Arg-301 and Glu-307, such that the interaction between Glu-307 and Arg-82 observed in zebrafish P2X4 was disrupted [Glu-310 and Arg-85, zfP2X4 numbering; compare Supplementary Figure S1B to Figure 4A). Conversely, in our induced-fit docking of Tian-13 (Figure 4C), the molecule is flipped compared with what was previously reported (Tian et al. (2020)]. Our docking positioned Tian-13 deeper in the pocket where the hydroxyl group of the chlorophenyl moiety established a hydrogen bond with Asp-88, modifying its orientation, whilst the 2,4-trifluoromethyl-phenyl moiety acted as a “wedge” at the top of the pocket (Figure 4C). Strikingly, our docking shows how NP-A, one of the most potent antagonists discovered to date, may occupy nearly the full length of the allosteric pocket, with the di-benzo scaffold making a potential interaction at the top of the pocket, disrupting the interaction between Glu-307 and Arg-82 (Figure 4B). The chlorophenyl moiety was positioned at the base of the pocket with the chloride making a polar interaction with the Ala-297 backbone, whilst the phenyl sulphonamide linker made further multiple interactions with the Ala-87 backbone and Tyr-299 (Figure 4B). Overall, we obtained poses fitting deeper in the allosteric pocket for most of the ligands with the highest potency (e.g., Mahmood-9g, Mahmood-9o, NP-1815-PX, NP-A, Tian-13; Figure 4; Supplementary Figure S1) which suggests that interaction with residues located at the bottom of the pocket (Asp-85, Ala-87, Asp-88, and Ala-297) is favorable for higher potency. Furthermore, the induced-fit dock allowed us to observe how the binding of the ligand might disrupt the complex hydrogen bond and ionic interaction network that stabilizes the protein and allows channel activation.

## 4 Discussion

In this work, we conjectured that the binding site in human P2X4 for allosteric antagonists may be the same pocket as that recently demonstrated in panda P2X7 (Karasawa & Kawate, 2016) and docked BX430 into this pocket in molecular models of human and rat P2X4. We demonstrated by mutagenesis and functional assay that exchange of a residue within this pocket, 312, by mutation, confers rat-like BX430 potency onto human P2X4 and *vice versa*, results that strongly support those of (Ase et al., 2019). The simplest interpretation of our data is that BX430 binds to this allosteric pocket and that amino-acid residue 312 is directly involved in BX430 binding.

Ase et al. (2019) were also able to dock BX430 into the putative allosteric pocket in which residue 312 resides, producing a binding pose similar to that which we obtained shown in Figure 2. However, in our docking studies, we found that the conformation of Lys-298 was very important in governing the overall conformation of the pocket. When applying an induced-fit docking protocol, which simulates pocket amino acid sidechain flexibility, we observed a binding pose situated deeper within the pocket (shown in Figure 2B) with Asp-88 as key residue for interactions. While our data and that of (Ase et al., 2019) are in very close agreement, an alternative potential binding pocket for BX430 on the dorsal fin region (forming interactions with Asn-208, Ile-209, and Asp-224), based on mutagenesis studies using receptor chimeras, has recently been reported (Weinhausen et al., 2022). This data may indicate more than one potential binding site for BX430, although we did not observe BX430 occupying this region of the protein in our blind docking simulations.

Recent work by Bidula et al. (2022) has also investigated the allosteric pocket in human P2X4, discovering key amino acid residues involved in the binding of the negative allosteric antagonist 5-BDBD, and docking 5-BDBD into the pocket. The authors found evidence for 5-BDBD occupying a hydrophobic pocket (similar to that occupied by the isopropyl phenyl moiety of BX430 in our docking study), with additional interactions between the sidechains of Arg-301 and Tyr-300 and the carbonyl and amide groups of 5-BDBD. Our docking produced a similar conformation when the induced fit protocol was employed; however, the amide group of 5-BDBD was stabilised by Arg-301 and Glu-307 (Supplementary Figure S1B). Bidula et al. (2022) were unable to dock 5-BDBD into a molecular model of the open human P2X4 receptor, and when comparing the antagonist effects of 5-BDBD and BX430, found that BX430, but not 5-BDBD, was capable of blocking the open channel. The authors interpreted this finding to mean that in the open channel, access to the allosteric pocket is restricted for 5-BDBD. Our docking was performed with models of the closed state, but the extensive interactions that we suggest BX430 makes deep within the pocket may explain its continued ability to access the pocket in the open channel state.

Our analysis of the docking of other known P2X4 antagonists into the receptor extracellular domain using blind docking simulations indicated the BX430 binding pocket as a highly favoured cavity for many of the antagonists, and in addition there was a good correlation between the estimated binding energy of the blind docking and the potency of the compounds. This data suggests that many of the antagonists bind in the BX430 allosteric pocket. It is important to state here that our *in silico* findings have not been experimentally validated using receptors bearing mutations within the allosteric pocket. Strikingly, we were unable to successfully dock PSB-12054 into the allosteric pocket, suggesting that PSB-12054 (and the related PSB-12062) may bind to a different pocket on human P2X4, or only be able to bind to the open conformation of the receptor.

In our induced fit docking analysis, we found that all the ligands interacted with one or more of a network comprised of Arg-82, Asp-85, Ala-87, Asp-88, Trp-164, Ala-297, Glu-307, and Arg-309. This network of amino-acids forms critical interactions important for the structure and activity of human P2X4 (Zhao et al., 2016), particularly the salt bridge formed between Asp-88 and Arg-309, which is stabilised by Trp-164. We observed in several instances that the interactions among this amino-acid network were substantially disrupted. This was particularly true for compounds which display high potency, where we obtained poses of the ligands where they were positioned deeper into the allosteric pocket. In the case of Mahmood 4n and Tian-13, we obtained docking poses that are flipped compared to what has been published [Mahmood et al. (2022a); Tian et al. (2020)]. In these cases, the hydrophobic moieties (adamantine and the 2,4-trifluoromethyl group for Mahmood 4n and Tian-13, respectively) were positioned deeper in the pocket, indicating that there may be a need for conformational rearrangement in order to accommodate such a hindering group, and that this might cause the disruption of the key amino acid interaction network. We would expect that these disruptions would substantially impair the ability of human P2X4 to undergo ATP-induced conformational change when ligands are bound in the allosteric pocket.

It is difficult to infer information about the selectivity of the P2X4 antagonists from our studies, as we focused solely on modelling binding to the allosteric pocket of a molecular model of human P2X4. However, in their structure-activity relationship studies, Tian et al. (2020) showed that the hydroxyl of the chlorophenol moiety of Tian-13 is essential for antagonist activity but not responsible for selectivity (Figure 4C). A number of compounds developed by Tian et al. (2020) show submicromolar potency to P2X1, P2X4, and P2X7 [Tian et al. (2020)]. From our docking of Tian-13, we observe that the hydroxyl group in question interacts with the backbone of Asp-88, and we interpret this to mean that an interaction with Asp-88 is desirable for antagonist activity. As Asp-88 is conserved across the P2X receptor family, and forms a critical salt bridge interaction, this may partially explain why this series of compounds also has activity at P2X1 and P2X7, and means that the selectivity of Tian-13 will need to be developed *via* interaction with non-conserved residues.

It is interesting to note that, even though is it not among the most potent P2X4 antagonist, in our docking BX430 occupies a position deep within the allosteric pocket. This may suggest that not only deep positioning within the pocket, but also some more bulky or hindering moiety might be necessary to efficiently disrupt the key structural network.

In summary, we have used a combination of molecular docking, mutagenesis and functional assay to demonstrate the likely binding pocket for the allosteric antagonist BX430 in human P2X4, which confirms the work of (Ase et al., 2019), but also suggests a distinct, deeper positioning of BX430 where it forms key interactions including with Asp-88. Furthermore, we have performed molecular docking on a series of potent P2X4 antagonists in the allosteric pocket, enabling us to estimate binding energies and identify disruption to a key structural network of amino acids as a main determinant of high antagonist potency. Our findings will be useful for the future design and development of allosteric P2X4 antagonists with increased potency.

## Data Availability

The raw data supporting the conclusion of this article will be made available by the authors, without undue reservation.
