# Supplementary material for: Identification of the molecular determinants of antagonist potency in the allosteric binding pocket of human P2X4
Source: Front Pharmacol. 2023 Feb 9;14:1101023. doi: 10.3389/fphar.2023.1101023 (PMC9947563; doi:10.3389/fphar.2023.1101023)
Supplement: Supplementary file 1 [file DataSheet1.docx]

Supplementary Material

# Supplementary Figure

**Supplementary Figure 1.** Induced-fit docking of Sunovion-A (A), 5-BDBD (B), BAY-1797 (C), indophagolin (D), Mahmood-4n (E), Mahmood-9o (F), Mahmood-9g (G), and NP-1815-PX (H) into a model of human P2X4. Dashed lines indicate polar interactions.
